# Structural elucidation and physicochemical properties of mononuclear Uranyl(VI) complexes incorporating dianionic units

**DOI:** 10.1038/srep32898

**Published:** 2016-09-06

**Authors:** Mohammad Azam, Gunasekaran Velmurugan, Saikh Mohammad Wabaidur, Agata Trzesowska-Kruszynska, Rafal Kruszynski, Saud I. Al-Resayes, Zeid A. Al-Othman, Ponnambalam Venuvanalingam

**Affiliations:** 1 Department of Chemistry, College of Science, King Saud University, P. O. Box 2455, Riyadh 11451, KSA; 2 School of Chemistry, Bharathidasan University, Tiruchirappalli–620024, Tamil Nadu, India; 3 Institute of General and Ecological Chemistry, Lodz University of Technology, Zeromskiego 116, 90-924, Lodz, Poland

## Abstract

Two derivatives of organouranyl mononuclear complexes [UO_2_(L)THF] (1) and [UO_2_(L)Alc] (2), where L = (2,2′-(1E,1′E)-(2,2-dimethylpropane-1,3-dyl)bis(azanylylidene, THF = Tetrahydrofuran, Alc = Alcohol), have been prepared. These complexes have been determined by elemental analyses, single crystal X-ray crystallography and various spectroscopic studies. Moreover, the structure of these complexes have also been studied by DFT and time dependent DFT measurements showing that both the complexes have distorted pentagonal bipyramidal environment around uranyl ion. TD-DFT results indicate that the complex 1 displays an intense band at 458.7 nm which is mainly associated to the uranyl centered LMCT, where complex 2 shows a band at 461.8 nm that have significant LMCT character. The bonding has been further analyzed by EDA and NBO. The photocatalytic activity of complexes 1 and 2 for the degradation of rhodamine-B (RhB) and methylene blue (MB) under the irradiation of 500W Xe lamp has been explored, and found more efficient in presence of complex 1 than complex 2 for both dyes. In addition, dye adsorption and photoluminescence properties have also been discussed for both complexes.

The chemistry of uranium is dominated by hexavalent uranyl dication (UO_2_^2+^) which is a linear triatomic species capped with terminal oxygen atoms^1^,^2^. The uranyl ion is remarkably stable form of natural uranium which exists in nuclear fuel processing, and usually exhibits tetragonal, pentagonal, and hexagonal bipyramidal geometries in the equatorial plane^3^,^4^. Furthermore, uranyl dication shows little propensity to involve in various reactions characteristic of its group 6 transition-metal analogues [MO_2_]^2+^. [M = Cr, Mo, W]^5^. It is worth mentioning that the transition metal analogues of uranyl dication always adopt bent geometry whereas the uranyl ions are essentially linear with bond angle close to 180°, and are strongly covalent in nature. Moreover, the uranyl ion is almost always found with 4, 5 or 6 ligands coordinated to the uranyl cation in the equatorial plane. Interestingly, the bond lengths to the equatorial ligands are always longer than to the axial uranyl oxygen^6^. However, the uranyl complexes with cyclopentadienyl-based ligands exhibit coordination number upto 1–3^7^.

Salen ligands form neutral complexes with uranyl ion UO_2_^2+^ in tetradentate fashion in which a fifth coordination site is occupied by additional monodentate ligand such as hard anion or neutral donor molecule or a solvent molecule in equatorial position^8^. Interestingly, the presence of solvent molecule at the fifth position in equatorial plane plays a significant role in the activation of the substrate in catalysis^8^. Furthermore, Uranyl(VI) complexes exhibit various promising physicochemical properties, such as photoluminescence, photocatalysis, and photochemical reactivity^9^. The photoluminescence in uranyl complexs is due to the excitation and relaxation of the UO_2_^2+^ group. In some cases, the interaction between the ligands and the uranium centers via the “antenna effect” also causes photoluminescence^10^. In addition, uranyl complexes have also been reported to have some rare properties like pollutant adsorption properties because of their large surface area and functional groups^11^.

Herein we are reporting the structure investigations of two derivatives of salen ligand based organouranyl mononuclear complexes [UO_2_(L)THF] (1) and [UO_2_(L)Alc] (2) supported by single crystal X-ray diffraction measurements and various spectroscopic studies. Furthermore, to make a deeper understanding, a comprehensive computational study on the electronic structure and bonding has also been carried out using DFT calculations. The photocatalytic activity of these complexes have also been investigated and found that both the complexes exhibit remarkable photocatalytic activity on the degradation of rhodamine B (RhB) and methylene blue (MB) dyes. In addition, we have also studied their adsorption and photoluminescent applications.

## Results

The complexes 1 and 2 were prepared via a straightforward interaction of salen ligand^12^ and uranyl acetate in 1:1 molar ratio in THF and ethanol, respectively, producing deep coloured solvated organouranyl complexes. Both the complexes have similar slightly distorted pentagonal bipyramidal morphology around the uranium centre, which is confirmed by X-ray crystallography and various spectroscopic studies. In addition, we justified our experimental results by DFT calculations and TD-DFT measurements.

We have employed our complexes in photocatalysis and found them effective for the degradation of rhodamine-B (RhB) and methylene blue (MB). However, complex 1 found more effective in degradation of dyes in comparison of complex 2. Furthermore, we also tested the dye adsorption and photoluminescence properties of both the complexes.

## Discussion

### Crystal structure of organouranyl complexes 1and 2

**Figure 1:**
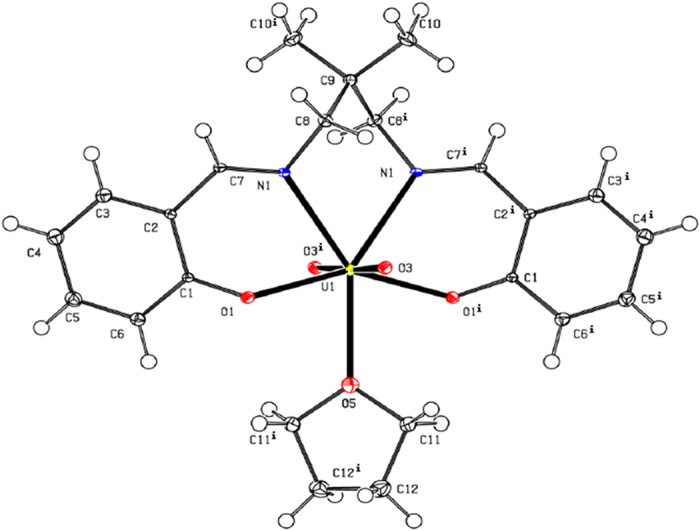
The molecular structure of complex **1** plotted with 50% probability of displacement ellipsoids.

**Figure 2:**
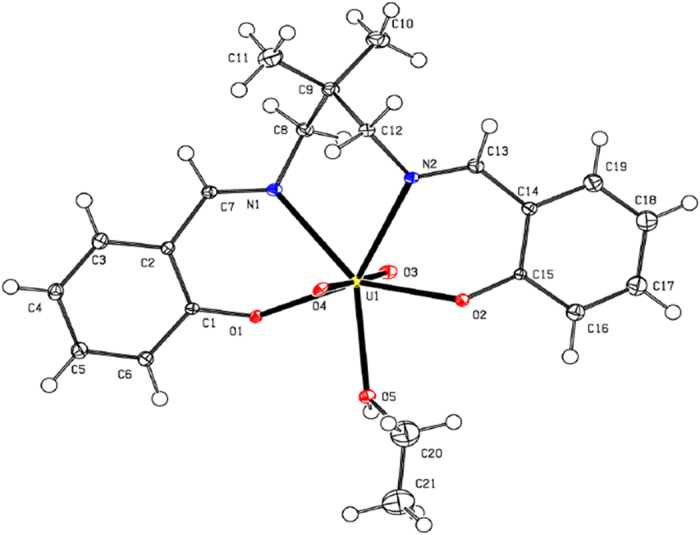
The molecular structure of complex **2** plotted with 50% probability of displacement ellipsoids.

**Figure 3:**
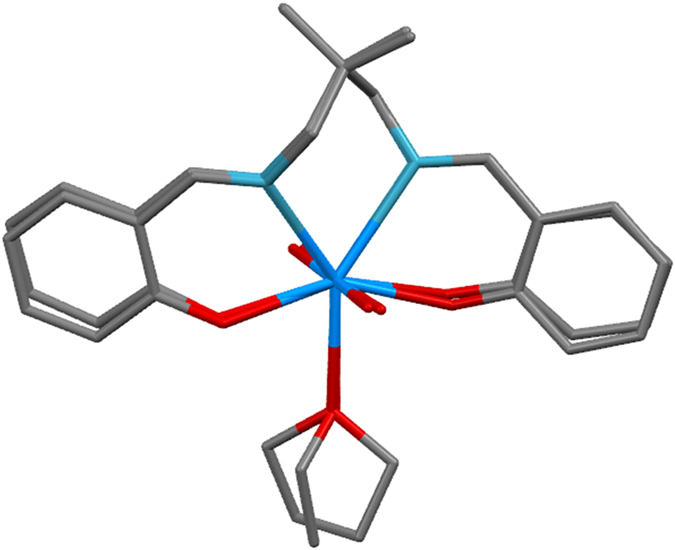
Superimposition of the molecular structure of complexes 1 and 2.

A perspective view of complexes 1 and 2 structure is shown in Figs 1 and 2, respectively. The compounds consists of complex molecules composed from the double deprotonated tetrafunctional chelating N,N′-bis(2-hydroxybenzylidene)-2,2-dimethyl-1,3-propanediamine ligand, the dioxouranium(VI) cation and the metal-coordinating solvent molecule (tetrahydrofuran and ethanol, respectively in complexes**1** and **2**). The U1, O5 and C9 atoms of complex **1** occupy special position e of *C*2/*c* space group with site symmetry 2 and multiplicity 4, thus one molecule is located in two asymmetric units. Additionally the two rotation fold axis cross the mid-point of the C12-C12^i^ bond (symmetry code: (i) −x, y, −z + 1/2) of complex **1**. All atoms of complex 2 occupy the general positions, however the chelating ligand and the dioxouranium(VI) cation possess the pseudosymmetry twofold rotation axis going through the U1 and C9, analogously this one of complex 1. Consequently the respect pairs of analogous atoms of (N,N′-bis(Salicylidene)-2,2-dimethyl-1,3-propanediaminato)-dioxouranium(VI) are related by non-crystallographic, above mentioned, pseudosymmetry element. The molecular symmetry of complex **2** is broken only by solvent molecule i.e. the ethanol molecule, which does not possess the internal symmetry of twofold rotation axis (in opposition to tetrahydrofuran). The (N,N′-bis(Salicylidene)-2,2-dimethyl-1,3-propanediaminato)-dioxouranium(VI) moiety in both complexes possess very similar conformations [Fig. 3], and the root mean squares deviation of superimposed moieties is 0.25(1) (the most distant equivalent C6 atoms are separated at 0.47(1) Å). The uranium atoms are seven-coordinated by two imine nitrogen atoms, two alkoxide oxygen atoms, two oxide oxygen atoms and one oxygen atom of solvent molecule. The coordination environment of the metal atoms can be described as slightly distorted pentagonal bipyramid^13^ with the oxide oxygen atoms located at the polyhedron apexes. The presented compounds are the first example of usage of the N’-bis(2-hydroxybenzylidene)-2,2-dimethyl-1,3-propanediamine as a building block for construction of coordination sphere with coordination number equal to 7. In all known compounds of this ligand the coordination number is 4 (4 compounds), 5 (11 compounds) and 6 (16 compounds)^14^, due to usage of metal ions with different radii. The salicylidene-1-methaneaminato moieties are distorted from planarity (observed in pure ligand 12) as a results of coordination to a relatively large uranium cation. The analysis of C-N bond lengths shows that the double bonds are fully localised within benzylideneamino moieties (Table 1).

The each tetrahydrofuran moiety of complex **1** is bonded to the two oxygen atoms of dioxouranium(VI) cationic core *via* weak C—H•••O intramolecular hydrogen bonds^15^ (Table 2) and these interactions form the C_2_^2^(8)C_2_^2^(8)[R_2_^2^(10)] motifs of lowest degree of the unitary graph set. The second and higher levels of the graph set are identical due to existence of only one structurally independent intermolecular interaction in the crystal net. The described chains extend along crystallographic [001] axis. The O—H•••O intermolecular hydrogen bonds of complex **2** join the molecules in the supramolecular dimers characterised by the R_2_^2^(4)] motifs of lowest degree of the unitary graph set and the dimers are assembled to the supramolecular layer (propagating along crystallographic (10-1) plane) *via* C—H•••O intramolecular hydrogen bonds (forming the C(5) and R_2_^2^(16) motifs of lowest degree of the unitary graph set). The possibility of existence of π•••π interactions in the studied complexes was excluded on the basis of large distance between the centroids of aromatic rings.

#### Spectroscopic Studies

IR spectra of organouranyl complexes show strong bands at 1650 cm^−1^ and 1645 cm^−1^ due to *υ*_(C=N)_ vibrations in complex 1 and complex 2, respectively (see supporting information Figure [S1])^16^. The appearance of bands at 581 cm^−1^ in complex 1 and 585 cm^−1^ in 2 is due to ν_(U–N)_ vibrations^16^. The medium intensity peak owing to ν_(C-O)_ vibration appears at 1205 cm^−1^ and 1204 cm^−1^ in complex 1 and 2, respectively^17^. Asymmetric stretching vibrations due to UO_2_^2+^ cation appears at 894 cm^−1^ and 905 cm^−1^ for complex 1 and complex 2, respectively^18^,^19^, and the remaining bands were found at their expected positions.

Elemental analyses and ESI-MS data are consistent with the formulation of the complexes and show intense molecular ion peak at m/z 665.53 for complex 1, and m/z 638.49 for complex 2 due to [M + Na]^+^ cation adduct.

^1^H- NMR spectra of both the organouranyl complexes showed multiplets at 6.66–7.65 ppm region due to aromatic proton. Aliphatic protons due to –CH_2_ of THF ring coordinated to the uranyl ion were observed at 1.34–3.59 ppm in complex 1, while complex 2 showed aliphatic protons due to coordinated ethanol at 1.90–3.47 ppm along with a singlet at 2.70 ppm (-OH) (see supporting information Figures [S2 and S3]), while the other protons were found at their expected positions. The ^13^C- NMR spectra showed a characteristic cluster of peaks between 120.3–134.3 ppm corresponding to the aromatic carbons. The signal due to –C-O carbon appears at 168.3 in complex 1 and 169.6 ppm in complex 2, respectively. Furthermore, ^13^C-NMR signals assigned to azomethine carbon appear at 168.3 ppm and 168.3 in complex 1, whereas it appears at 168.3 ppm in complex 2, while the rest of aliphatic carbon signals are in good agreement to their expected chemical shift values (see supporting information Figures [S4 and S5]).

The UV-Vis spectra of uranyl complexes 1 and 2, recorded in dichloromethane, show intense absorption bands in the visible region at 435.6 nm (ε = 9.8 × 105 M^−1^ cm^−1^) for complex 1 and λ_max_ 429 nm (ε = 1.6 × 105 M^−1^ cm^−1^) for complex 2, which are supposed to be due to f-d or charge-transfer transitions^20^,^21^,^22^.

To interpret the electronic spectra of the complexes, TD-DFT calculations were performed in dichloromethane solution (Table 3). The experimental results agree well with the calculated absorption spectra of complexes 1 and 2. The experimental band at 435.6 nm corresponds to the transition calculated at 458.7 nm originating from HOMO-1 → LUMO+3 transitions (98%) with LMCT character in complex 1 (Table 4), whereas in complex 2, the experimental band at 429 nm corresponds to the transition calculated at 461.8 nm which originates from HOMO-1 → LUMO+3 (61%) and HOMO-1 → LUMO+2 (22%) with significant LMCT character (Table 3).

#### Molecular geometry

**Figure 4:**
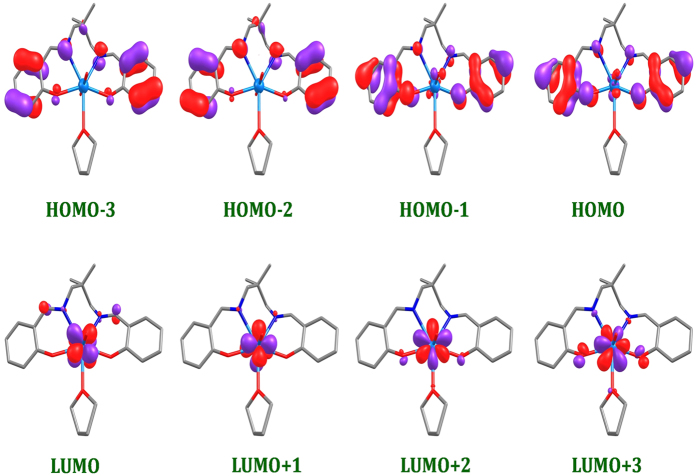
HOMO and LUMO orbitals of complex [UO_2_(L)THF] (1).

**Figure 5:**
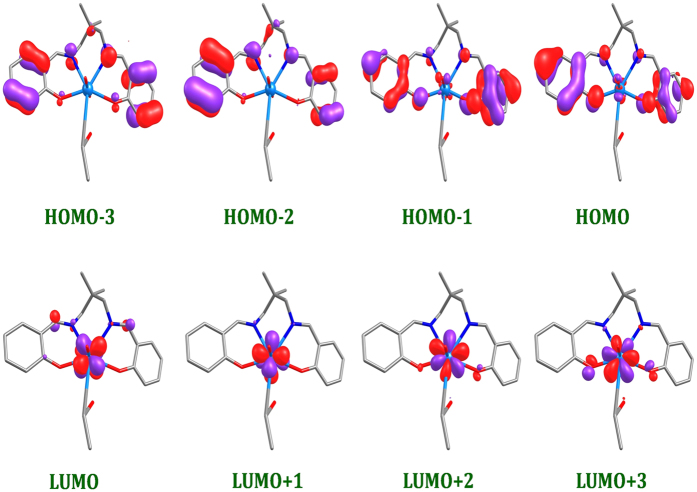
HOMO and LUMO orbitals of complex [UO_2_(L)C_2_H_5_OH] (2).

In order to get further insights into the electronic structure of these complexes, density functional theory (DFT) calculations at the B3LYP level were carried out. The optimized structures of complex 1 and 2 reveal that both the complexes have distorted pentagonal bipyramidal geometry (See Supporting information Figure [S6]). A comparison between the calculated and experimentally determined selected bond lengths and angles are listed in Table S1 (supporting information). The calculated geometries of both complexes agree well with experimental results. Both U = O3 and U = O4 bond distances are same i.e. 1.793 Å and are equal to previously reported density functional studies^23^. Furthermore, O3 = U = O4 angles in complex 1 and 2 were calculated to be 177.9° and 176.4°, respectively which is in agreement with the experimental value of 177°. The electronic and spectral properties of the complexes depend mostly on HOMO, LUMO and the band gap of the complexes. Therefore, the energy of the frontier molecular orbitals of the complexes 1 and 2 (from HOMO-3 to LUMO+3), and their gaps have been computed (Table 4). The HOMO and LUMO energy are found to be −5.38/−2.10 eV for complex 1, whereas they are calculated to be −5.53/−2.27 eV for complex 2, respectively. HOMO-LUMO gaps (Δ*E*_*H−L*_) were calculated as 3.27 eV and 3.26 eV for complex 1 and 2, respectively, suggesting that the f−f transitions in the electronic spectra of these complexes would be found at similar wavelengths. Interestingly, LUMO is localized on Uranium atom whereas HOMO on the ligand, L [Figs 4 and 5], suggesting that the unoccupied orbital of the complex is featured with U (f) character^23^.

The complex has been divided into four fractions namely; Uranium (U), Oxygen (2“O”), solvent (THF in complex 1; EtOH in complex 2), and Ligand (L), and their contributions towards FMOs have been computed by QMForge program^24^ (Table S2 supporting information). The molecular orbital diagram clearly state that the ligand fragments contributes about 93% towards the HOMO, whereas uranium fragment with 83% stabilize mostly LUMO. Furthermore, it is evident that the ligand fragment (92–99%) mostly contributes HOMO-1 to HOMO-3 whereas the LUMO+1 to LUMO+3 are localized on Uranium atom (82–90%) (See Supporting Information Figures [S6 and S7]). The change in the seventh coordinated solvent from complex 1 to 2 does not change order of FMO energies. However, change in the solvent from CH_3_OH^23^ to C_2_H_5_OH (Complex 2) doesn’t make any change in the FMO contributions, whereas the complex coordinating with the THF (Complex 1) have significant contribution to the HOMO and LUMO.

#### Energy Decomposition Analysis (EDA)

EDA reveals the nature of interactions between [UO_2_(L)] and solvent in a quantitative way based on the Ziegler’s scheme^25^. EDA calculations have been carried out to divide the seventh coordinated solvent (THF in complex 1; EtOH in complex 2) as one part and remaining fragment is another part (Table S3 supporting information). The complex 2 has (−28.91kJ mol^−1^) less negative bonding energy than for complex 1 (−73.71 kJ mol^−1^). The negative bonding energy for both the complexes is due to electrostatic interaction. Furthermore, the steric interaction (Pauli repulsion+electrostatic interaction) is larger in the complex 2 than in the complex 1 by 173 kJ mol^−1^. The orbital interaction is significantly more negative for the complex 2 than for the complex 1, thus making complex 2 more stable by 130 kJ mol^−1^. In fact, the difference in the stability is due to changes in the symmetry. EDA study reveals that the electrostatic interaction is greater than that of orbital interaction in both the complexes. Therefore, the interaction between the seventh coordinated solvent and other parts is mostly electrostatic (65%) (Table S4 supporting information).

#### Natural Bond Orbital (NBO) analysis

The lone pair on O3 and O4, and the anti-bonding lone pair orbital of U atom are involved in ground state stabilization of both the complexes through n_(O)_ → n*_(U)_ interactions. Furthermore, ground state stabilization is due to n_(N)_ → n*_(U)_ and n_(O5)_ → n*_(U)_ interactions. In addition, one of the lone pair, LP(1) interact with n*_(U)_. Supporting information Figure [S7] shows major orbitals involved in the second order perturbation interactions n_(O)_ → n*_(U)_ that stabilize the system

#### Photocatalytic studies

The photocatalysts have received tremendous interests worldwide due to their significant role in waste water management and air purifying as they thoroughly decompose the organic contaminants^26^. Among the various photocatalysts, the uranyl complexes have significantly been used for this purpose^27^. Therefore, our prepared uranyl complexes 1 and 2 were tested as the model substrate to evaluate the photocatalytic activity by observing the degradation of RhB and MB. The control experiments were conducted to avoid the possibility of degradation from molecular or oligomeric species that could be formed due to the dissolution of the solid samples in the reaction systems. The electronic spectra of dyes containing organouranyl complexes 1 and 2 showed that both the complexes display strong absorption in the UV/Vis region and the absorption intensities were gradually decreased with time after irradiation with 500W Xe lamp, indicating that both the complexes possesses photocatalytic property under the UV or visible irradiation. It is well reported that the typical UV/vis absorption spectra of uranyl complexes normally found in the UV region is due to the charge transfer electronic transition within U = O double bonds, while the absorption in the visible region is due to U_5f_ ← O_2p_ ligand to metal charge transfer (LMCT) transitions within the O atom of the coordinated ligand and the vacant orbital of uranium (VI) ions^26^,^28^.

**Figure 6:**
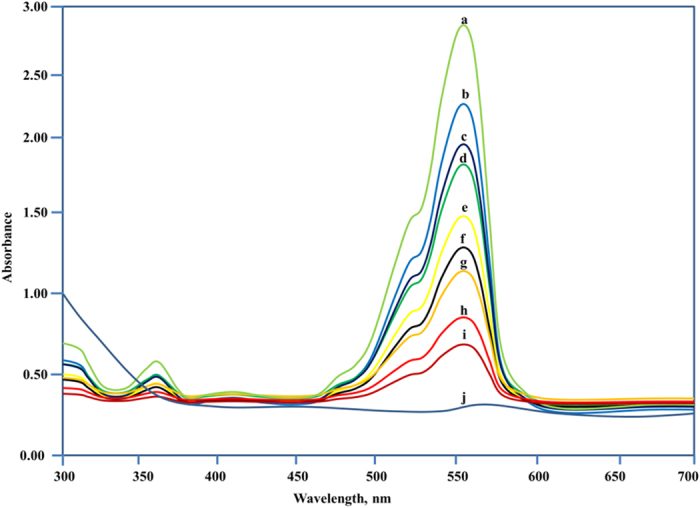
The UV–vis absorption spectra of the aqueous solution of RhB during the irradiation under visible light with complex 1 at time interval (a → 0; b → 10; c → 20; d → 30; e → 45; f → 60; g → 90; h → 120; I → 150; j → 180 min; the curve a is the control experiment without any catalyst.

**Figure 7:**
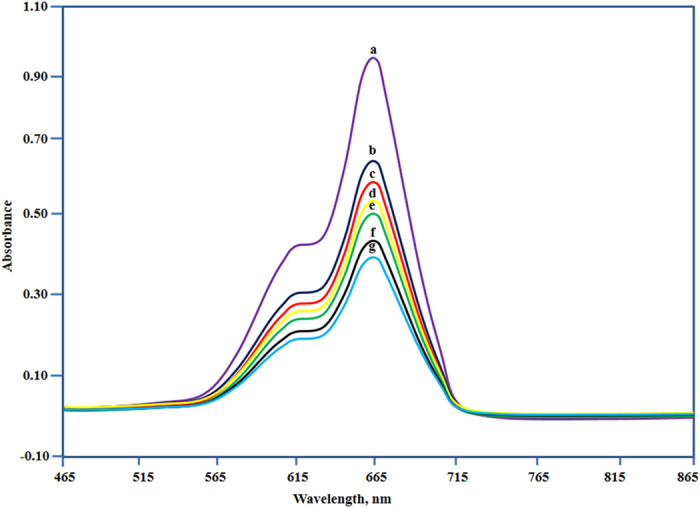
The UV–vis absorption spectra of the aqueous solution of MB during the irradiation under visible light with complex 1 at time interval (a → 0; b → 10; c → 30; d → 60; e → 90; f → 120; g → 180 min; the curve a is the control experiment without any catalyst.

The concentration changes of RhB (Rhodamine B) and MB (Methylene Blue) were described by different absorbance at a certain wavelength 554 nm and 664 nm, respectively. Complex 1 showed accelerated degradation of RhB (90%) when irradiated with visible light for 3 h [Fig. 6]. However, the degradation rate on addition of complex 2 was around 70% under similar conditions. On the other hand, the complex 1 shows remarkable decrease in intensity of absorbance when exposed to visible light, and brings approximately 58.5% degradation of MB within 3 h [Fig. 7]. However, degradation rate was not significant in case of complex 2 under similar conditions. We conclude that complex 1 exhibits high photocatalytic activity for both RhB and MB under visible light and could be useful for removal of pollutants from waste water. The differences in dye degradation properties of both complexes might be due to the presence of different coordinating solvents viz., tetrahydrofuran and alcohol in complexes 1 and 2, respectively. The presence of tetrahydrofuran in complex 1 may influence the electron abstraction from the dyes more than ethanol of complex 2. Therefore, the tendency of formation of ^**∙**^O_2_^−^ is much more for complex 2 than complex 1. Another possibility for the different extent of degradation for complexes 1 and 2 is supposed to be due to the dioxo uranyl moiety and lattice molecules^29^. To compare the results, the dyes were kept under UV irradiation without adding complexes and no such changes in the concentration of dyes were observed.

#### Photocatalytic mechanism

**Figure 8:**
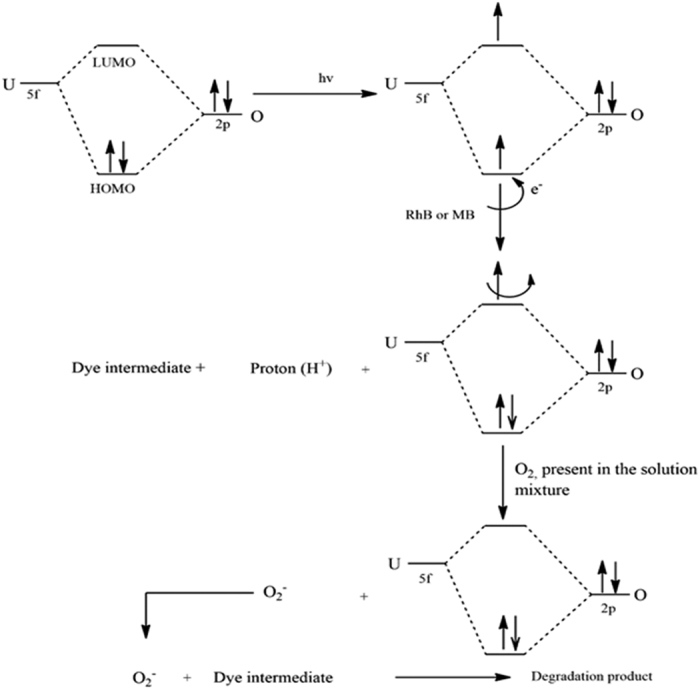
The possible degradation mechanism of dyes via Photoexcitation of UO_2_^2+^ species in organo-urnayl complexes.

The photocatalytic reactions mechanism of uranyl species involves electron transfer and hydrogen abstraction^30^. Once the complex irradiate under light, the electrons from the highest occupied molecular orbitals (HOMO) of UO_2_^2+^ group may be promoted to the lowest unoccupied molecular orbitals (LUMO) which is the empty nonbonding orbitals localized on the uranium atom and consequently an excited UO_2_^2+^ species (UO_2_^2+*^) was generated [Fig. 8]. The interaction between 5f orbitals of U and the symmetry match 2p group orbitals of O formed the bonding HOMO and same number of nonbonding LUMO. As the energy level of LUMO is higher, the excited electron in this orbital is unstable and tries to come back to the HOMO instantly. However, the presence of organic dyes within proper orientation and reasonable range, electron from the nucleophile groups of these molecules may be abstracted by the excited UO_2_^2+^ species and it occupied the HOMO and yields an intermediate and a proton. Hence, the possibility of returning of the excited electrons becomes challenging and it remain in the LUMO until it abstracted by other electronegative substances such as O_2_. Due to this, the highly reactive oxygen-centered radicals such as ^∙^O_2_^−^ or peroxide anion radical are formed and these are responsible to oxidize and decompose the intermediates formed in the solution and results complete degradation of the dyes [Fig. 8]. Both RhB and MB are stable to O_2_ and visible light. However, these dyes, RhB and MB, undergo degradation with proton abstraction and stepwise de-ethylation on association with photo-excited uranyl centers which results the formation of active intermediates of dyes. These intermediates are then further cracked by the oxygen-centered species in the solution and ending up with the formation of small organic acids and/or CO_2_^31^,^32^,^33^.

#### Fluorescence studies

**Figure 9:**
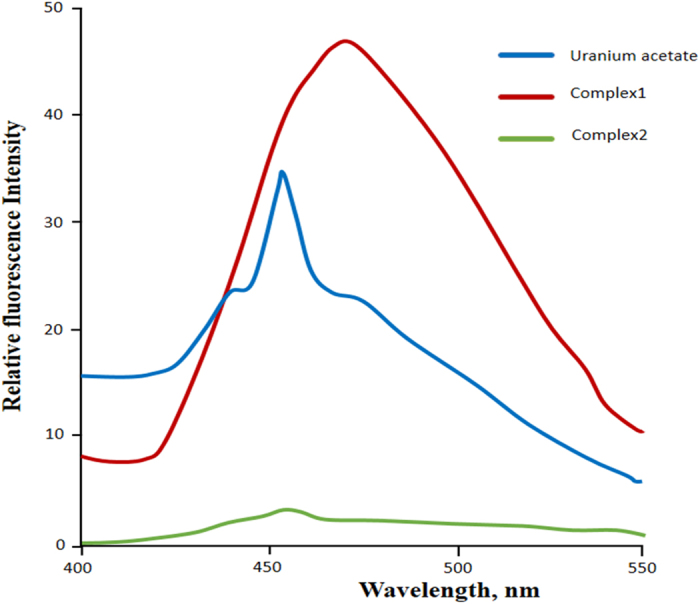
Fluorescence emission spectra of metal and complex 1 and complex 2. Concentration and excitation wavelength of metal: c = 5.5 104 M, λexc = 300 nm, 1: c = 6.3 105 M, λexc = 321 nm.

Fluorescence emission spectra of uranyl acetate and organouranyl complexes **1** and **2** have been studied at excitation wavelength of 399 nm with a slit width of 5 nm, while 10^−3^ M DMSO solution was used as solvent [Fig. 9]. It is obvious from the earlier research that fluorescence spectra of organouranyl complexes have characteristic six peaks relating to S_11_ → S_00_ and S_10_ → S_0_ν electronic transitions, where ν = 0–4^34^,^35^. The intense peak related to S_10_ → S_0_ν transition for uranyl acetate is observed at emission maxima 454 nm. The prominent peak for complex 1 is observed at emission maxima 472 nm and showing bathochromic shift of around 18 nm when compared to spectrum of uranyl acetate precursor. Furthermore, the fluorescence emission spectrum of complex 2 is relatively broad, and there is no significant shift on comparing with the emission spectrum of uranyl acetate precursor. Therefore, we assume that the changes in characteristic shape and emission maximum of the complexes occurs due to the coordination of uranyl(VI) central ion with the organic templates, since the emission that is observed from the uranyl complexes is actually ligand based^21^.

#### Adsorption properties studies

**Figure 10:**
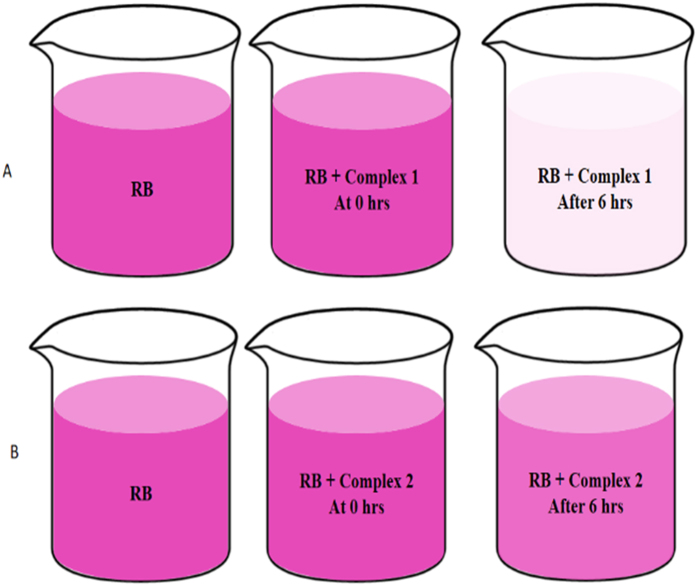
The photographs of dye absorption at different time interval. (**A**) Pure Rhodamine B and Rhodamine B with Complex 1 at 0 hrs and after 6 hrs (**B**) Rhodamine B and Rhodamine B with Complex 2 at 0 hrs and after 6 hrs time interval.

In order to explore the adsorption properties of the synthesized complexes, batch adsorption experiments were conducted for the removal of target dye. For this purpose, 0.005g each complex was taken in separate 200mL conical flask and 50 mL dye solution of initial concentration 50 mg L^−1^ were added in each flask and kept on a shaker at 100rpm speed. The factor influencing the adsorption efficiency, such as solution pH was taken in consideration and adjusted using NaOH and HNO_3_ solutions. After, certain time intervals (0, 60, 120, 150, 240 and 360 min), the dye solution mixtures was centrifuged at 5000 rpm for 5 min and the concentration of residual RB dye was determined with an UV–vis spectrophotometer (U-3010) at the maximum wavelength of 544 nm. The results obtained after 6 h of adsorption by UV spectra indicates that almost 90% dye was removed due to adsorption with the synthesized complex from the aqueous solution [Fig. 10].

The adsorption capacity qt (mg g^−1^) and removal rate (Removal %) was calculated according to the following equations:


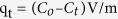



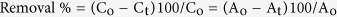


where, C_o_ and C_t_ (mg L^−1^) were the concentration of the dye at initial and time t, respectively. A_o_ and A_t_ represented the absorbance of Rhodamine B before and after the adsorption. V (mL) was the volume of the RB solution and m(mg) was the mass of adsorbents.

**Figure 11:**
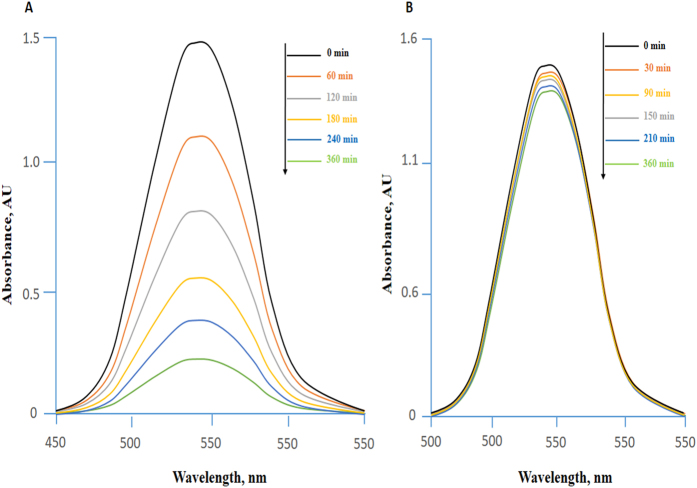
The dye absorption spectra at different time interval. (**A**) Rhodamine B with Complex 1 and (**B**) Rhodamine B with Complex 2 at different time interval.

The results indicates RhB was almost completely removed from aqueous solution after 6 h of adsorption with complex 1, as very low absorbance was detected in UV spectra [Fig. 11A]. In case of complex 2 with same dye the results of adsorption were not satisfactory (Fig. 11B]. This might be due to the less tendency of donating the lone pair of electrons of oxygen of ethanol in complex 2 compared to oxygen of THF in complex 1. Therefore, complex 1 easily absorb the dye by electrostatic interaction with the quaternary nitrogen atom [=N^+^ (C_2_H_5_)_2_] of the RhB. The advantages for the adsorption using these complexes is that the water insoluble nature of them makes it very easy to separated them from the reaction systems and allow the recycling and re-use of them.

In conclusion, we have reported the structure investigation of two derivatives of mononuclear organo-uranyl complexes with the formula, [UO_2_(L)THF] (1) and [UO_2_(L)C_2_H_5_OH] (2). Both the complexes around uranium center have slightly distorted pentagonal bipyrimidial geometry. Furthermore, the structure and bonding properties of the complexes are investigated by density functional theory. FMO analysis reveals that the ligand contributes more towards HOMO whereas LUMO is mainly stabilized by Uranium. The outcome from TD-DFT indicates a band appears at 458.7 nm that arises due to HOMO-1 → LUMO+3 (98%) transition associated with the LMCT character for complex 1, whereas complex 2 shows a band predicted at 461.8 nm have a contribution from HOMO-1 → LUMO+3 (61%) and HOMO-1 → LUMO+2 (22%) transition with significant LMCT character. EDA analysis reveals that complex 2 is more stable by 130 kJ mol^−1^ than complex 1. The results obtained from NBO analysis confirm that stability of the complexes in ground state is mostly due to n → n* interaction. Moreover, various physicochemical properties of complexes 1 and 2 has been studied and found complex 2 effective photocatalyst when tested against RhB and MB. However, complex 1 showed significant photoluminescence property in DMSO. Moreover, dye adsorption properties of both complexes has also been checked and found that complex 1 is more effective than complex 2 for the adsorption of dye (RhB).

## Methods

### Materials and instrumentation

All reagents and solvents were procured from commercial sources and used as received. C,H,N analyses were carried out on ElementarVarrio EL analyzer. ^1^H and ^13^C NMR spectra were recorded on JEOL spectrometer at 400 MHz (^1^H-NMR) and 100 MHz (^13^C-NMR), respectively. The chemical shifts (δ in ppm) were reported downfield from tetramethylsilane (TMS, δ scale) with d_6_-DMSO resonance referenced as the internal standard. FT-IR spectra were obtained on Perkin Elmer 621 spectrophotometer at 400–4000 cm^−1^. Fluorescence measurements were recorded on a Shimadzu Spectro Fluorophotometer (model RF5301PC) equipped with RF 530 XPC instrument control software using a quartz cell of 1 cm path length. ESI-MS analyses were performed using Micromass Quattro Premier Tandem MS.

### Synthesis of organouranyl complexes

#### Synthesis of complex 1, [UO_2_(L)THF].

(UO_2_(CH_3_COO)_2_.2H_2_O) (0.46 g, 1.1 mmol) dissolved in minimum quantity of THF (10 mL) was added slowly to a stirred solution of ligand, L^12^ (0.38 g, 1.1 mmol) in THF (20 mL) at room temperature. The reaction mixture was stirred for 10 hours to yield orange colored solution. The solvent of the reaction mixture was removed under vacuum and the product was washed with diethyl ether and hexane followed by re-crystallization in tetrahydrofuran. Orange colored crystals were separated out in a week.

Yield: 1.09 g (74.4%). Anal.Calc. for C_23_H_28_N_2_O_5_U C, 43.31; H, 4.69; N, 4.21.Found: C, 43.35; H, 4.72; N, 4.25%. FT-IR (cm^−1^, KBr), 1630 (-CH = N), 581 ν(U–N), 894 ν (U = O), ^1^H NMR (400 MHz, d_6_-DMSO): δ (ppm) 9.23 (s, –CH = N), 8.53 (s –CH = N), 7.65 (d, 2H, H1, ^3^J_H-H_ = 6.6 Hz), 7.34 (dd, 2H, H2, ^3^J_H-H_ = 6.6 Hz), 6.94 (dd, 2H, H3, ^3^J _H-H_ = 6.6, ^4^J _H-H_ = 1.48), 6.67 (2H, H4, ^3^J _H-H_ = 6.6), 1.24 (s –CH_3_), 3.88 (s -CH_2_), 3.60 (m -CH_2_), 2.07 (s -CH_2_), 1.24 (s –CH_3_), 1.89 (s -CH_2_)

#### Synthesis of complex 2 [UO

_*2*_*(L).C*_*2*_*H*_*5*_*OH].* An ethanol solution (5 mL) of uranyl acetate (0.38 g, 0.9 mmol) was added to the solution of ligand, L^12^ (0.31 g, 0.9 mmol) in ethanol (15 mL) with constant stirring for 5 h resulting into orange colored solution. The solution is allowed to evaporate at room temperature. Orange colored crystals were isolated in few days.

Yield: 0.89 g (79.2%). Anal. Calc. for C_21_H_26_N_2_O_5_U: C, 40.39; H, 4.20; N, 4.49. Found: C, 40.42; H, 4.25; N, 4.55%. FT-IR (cm^–1^, KBr), 1623 (-CH = N), 585 ν(U–N), 905 ν (U = O), ^1^H NMR (d_6_-DMSO): δ (ppm) : 9.23 (s, 2H, –CH = N), 7.53 (dd, 2H, H2 ^3^J_H-H_ = 6.6 Hz, ^4^J_H-H_ = 0.72 Hz), 6.94 (dd, 2H, H3, ^3^J_H-H_ = 6.6 Hz, ^4^J_H-H_ = 0.72 Hz), 7.65 (d, 2H, H1 ^3^J_H-H_ = 7.32 Hz), 7.69 (2H, H4 ^3^J_H-H_ = 7.32 Hz), 1.23 (s, 6H, -CH_3_), 3.89 (s, 4H, -CH_2_), 2.49 (s, -**H**O-CH_2_CH_3_), 3.47–3.42 (qr, 2H, J = 11Hz, J = 7.32 Hz), 1.09–0.98 (t, 3H, J = 7.32 Hz, J = 6.6 Hz).

#### Computational details

Quantum chemical calculations were performed using the Gaussian09 program^36^. The hybrid B3LYP density functional^37^,^38^,^39^, and the Stuttgart RSC 1997 effective core potential (ECP) were used to explain the uranium atoms^40^,^41^. The pseudopotential represents 60 core electrons in uranium while the remaining 32 electrons are represented by the associated valence basis set. Carbon, hydrogen, oxygen and nitrogen atoms were described using 6-31G(d,p) basis set. On the basis of the optimized ground state geometries, the electronic transitions in the dichloromethane (CH_2_Cl_2_) solution were also calculated by time-dependent DFT^42^ at the same level using a polarized continuum model (PCM). Natural bond orbital (NBO) analysis was recorded by Gaussian09^43^,^44^. BP86/TZP with ZORA using ADF software has been used in Energy decomposition analysis^45^,^46^ to obtain various interaction energy components between the fragments by the following equation:


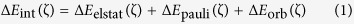


#### Crystal structure determination

The orange prism crystals of complexes 1 and 2 were mounted on a KM-4-CCD automatic diffractometer equipped with CCD detector, and used for data collection. X-ray intensity data were collected with graphite monochromatedCu*K*_α_ (λ = 1.54178 Å) radiation at temperature 100.0(1) K, with *ω* scan mode. The 6 seconds exposure time was used in both measurements, and reflections inside Ewald sphere were collected up to *θ* = 67°. The unit cell parameters were determined from 981 and 1657 strongest reflections, respectively for 1 and 2. Details concerning crystal data and refinement are given in Table 5. During the data reduction the Lorentz, polarization and numerical absorption^47^ corrections were applied. The structures were solved by partial structure expansion procedure. All the non-hydrogen atoms were refined anisotropically using full-matrix, least-squares technique on F^2^. All the hydrogen atoms were found from difference Fourier synthesis after four cycles of anisotropic refinement, and refined as “riding” on the adjacent atom with geometric idealisation after each cycle of refinement and individual isotropic displacement factors equal 1.2 times the value of equivalent displacement factor of the parent non-methyl carbon atoms, and 1.5 times of parent methyl carbon atoms. The methyl groups were allowed to rotate about the local three-fold axes. The XS, XL and XTL^48^ software was used for all the calculations. Atomic scattering factors were those incorporated in the computer programs. Selected interatomic bond distances and angles are listed in Table 1 and intermolecular interactions are listed in Table 2.

#### Analyses of photocatalytic activity

The photocatalytic activity of the complexes 1 and 2 was assessed by the degradation of rhodamine B (RhB) and methylene blue (MB) dyes under the influence of visible light irradiation. 500W Xe lamp with main output >400 nm was used as UV light source. During the experiment, 20 mg of each complex was suspended to the 10 ppm 100 ml aqueous solution of RhB and MB, respectively. The suspended solution was magnetically stirred for at least half an hour in the dark to establish adsorption/desorption equilibrium of dyes on the sample surface followed by exposure to 500W Xe lamp for irradiation with continuous stirring. After a certain time irradiation intervals, a series of certain amount of solutions were taken out from the reaction cell and collect them followed by centrifugation. The separated liquid supernatants were then subjected to spectroscopic analysis on the UV/vis spectrometer, and the dyes solutions were diluted as required. The absorbance of RhB was checked at absorption maxima 554 nm, while the MB was measured at 664 nm.

#### Fluorescence measurements

The fluorometric studies of uranyl acetate and organouranyl complexes 1 and 2 have been performed on Shimadzu spectro Fluorophotometer (model RF5301PC) fitted with RF 530 XPC instrument control software using a quartz cell of 1 cm path length. The excitation wavelength was chosen at 399 nm for all cases and the slit width was fixed at 5 nm. During the fluorescence experiment, the complexes were dissolved in 10^−3^ M DMSO and the data were recorded.

## Additional Information

**How to cite this article**: Azam, M. *et al.* Structural Elucidation and Physicochemical Properties of Mononuclear Uranyl(VI) Complexes incorporating Dianionic units. *Sci. Rep.* **6**, 32898; doi: 10.1038/srep32898 (2016).

## Supplementary Material

Supplementary Information

## Figures and Tables

**Table 1:** Selected structural data of compound **1** and **2** [Å, °].

Compound 1		Compound 2	
U1—O3	1.793(4)	U1—O3	1.781(3)
U1—O1	2.247(4)	U1—O4	1.781(3)
U1—O5	2.484(6)	U1—O2	2.230(3)
U1—N1	2.586(4)	U1—O1	2.305(3)
N1—C7	1.282(7)	U1—O5	2.447(3)
N1—C8	1.461(7)	U1—N1	2.566(5)
		U1—N2	2.573(4)
		C7—N1	1.283(7)
		N1—C8	1.470(6)
		C12—N2	1.468(6)
		N2—C13	1.284(7)
O3—U1—O3^i^	177.0(2)	O3—U1—O4	177.05(15)
O3—U1—O1^i^	88.19(17)	O3—U1—O2	88.68(15)
O3—U1—O1	92.59(17)	O4—U1—O2	94.02(14)
O1—U1—O1^i^	149.21(18)	O3—U1—O1	87.25(14)
O3—U1—O5	91.48(10)	O4—U1—O1	90.98(14)
O1—U1—O5	74.61(9)	O2—U1—O1	150.18(12)
O3—U1—N1	85.10(15)	O3—U1—O5	93.68(14)
O1—U1—N1	70.19(13)	O4—U1—O5	88.15(14)
O5—U1—N1	144.41(9)	O2—U1—O5	75.36(12)
O3—U1—N1^i^	92.49(15)	O1—U1—O5	75.45(12)
O1—U1—N1^i^	140.41(13)	O3—U1—N1	88.30(15)
N1—U1—N1^i^	71.19(18)	O4—U1—N1	88.87(15)
		O2—U1—N1	140.62(12)
		O1—U1—N1	68.76(12)
		O5—U1—N1	144.02(12)
		O3—U1—N2	91.39(14)
		O4—U1—N2	88.42(14)
		O2—U1—N2	70.38(13)
		O1—U1—N2	139.22(12)
		O5—U1—N2	145.21(12)
		N1—U1—N2	70.46(13)

Symmetry transformations used to generate equivalent atoms: (i) −x, y, −z + 1/2.

**Table 2:** Hydrogen bonds geometry of complexes 1 and 2 [Å, °].

D—H•••A	d(D-H)	d(H•••A)	d(D•••A)	<(DHA)
Compound **1**				
C11—H11A•••O3^i^	0.99	2.55	3.283(8)	130
Compound **2**				
O5—H5O•••O1^ii^	0.81	1.80	2.611(4)	180
C13—H13•••O4^iii^	0.95	2.54	3.470(6)	168
C18—H18•••O3^iv^	0.95	2.48	3.329(7)	148

Symmetry transformations used to generate equivalent atoms: (i) −x, −y, −z; (ii) 1 − x, 2 − y, 1 − z; (iii) 1/2 − x, −1/2 + y, 3/2 − z; (iv) 1−x, 1 − y, 1 − z.

**Table 3:** Calculated absorptions of complexes 1 and 2 in CH_2_Cl_2_.

Complexes	Cal (λ_max_) nm	Oscillator Strength (*f*)	E (eV)	Major Contribution (%)	Exp. (λ_max_) nm
Complex 1	458.7	0.0043	2.70	HOMO-1 → LUMO+3 (98%)	435.6
Complex 2	461.8	0.0013	2.68	HOMO-1 → LUMO+3 (61%)	429.0
				HOMO-1 → LUMO+2 (22%)	
				HOMO → LUMO+3 (12%)	

**Table 4:** Frontier molecular orbital energy (eV) of complexes 1 and 2.

	HOMO-3	HOMO-2	HOMO-1	HOMO	LUMO	LUMO+1	LUMO+2	LUMO+3	Δ*E*_*H−L*_
Complex 1	−6.56	−6.52	−5.44	−5.38	−2.10	−2.02	−1.89	−1.74	3.27
Complex 2	−6.69	−6.66	−5.62	−5.53	−2.27	−2.17	−2.09	−1.94	3.26

**Table 5:** Crystal and structure refinement data of complexes 1 and 2.

Compound	1	2
Empirical formula	C_23_H_28_N_2_O_5_U	C_21_H_26_N_2_O_5_U
Formula weight	650.50	624.47
Crystal system, space group	monoclinic, *C*2/*c* (No.15)	monoclinic, *P*2_1_/_n_ (No. 14)
Unit cell dimensions [Å, °]	*a* = 17.2330(15)	*a* = 11.4887(14)
	*b* = 13.5930(11)	*b* = 12.522(2)
	*c* = 11.8320(9)	*c* = 15.1669(17)
	*β* = 125.429(12)	*β* = 92.092(10)
Volume [Å^3^]	2258.4(5)	2180.5(5)
Z, Calculated density [Mg/m^3^]	4, 1.913	4, 1.902
*F(000)*	1248	1192
Crystal size [mm]	0.090, 0.087, 0.086	0.091, 0.090, 0.088
*θ* range for data collection [°]	4.53 to 72.37	4.58 to 72.47
Index ranges	−21≤*h*≤21, −16≤*k*≤16, −14≤*1*≤13	−14≤*h*≤14, −15≤*k*≤14, −18≤*1*≤18
Reflections collected/unique	12136/2242 [R_*(int)*_ = 0.0351]	23037/4309 [R_*(int)*_ = 0.0369]
Completeness [%]	100 (to *θ* = 67°)	99.9 (to *θ* = 67°)
Data/restraints/parameters	2242/0/144	4309/0/265
Goodness-of-fit on *F*^2^	1.093	1.089
Final *R* indices [*I* > 2σ(*I*)]	*R*1 = 0.0342, *wR2* = 0.0772	*R*1 = 0.0328, *wR2* = 0.0872
R indices (all data)	*R*1 = 0.0342, *wR*2 = 0.0772	*R*1 = 0.0330, *wR*2 = 0.0873
Largest diff. peak and hole [e•Å^−3^]	1.016, −1.057	1.921, −1.963

## References

[b1] Szigethy G. & Raymond K. N. Hexadentate Terephthalamide (bis-hydroxypyridinone) Ligands for Uranyl Chelation: Structural and Thermodynamic Consequences of Ligand Variation (1). J. Am. Chem. Soc. 133, 7942–7956 (2011). 21542587 10.1021/ja201511u

[b2] Hagberg D., Karlstrom G., Roos B. O. & Gagliardi L. The Coordination of Uranyl in Water: A Combined Quantum Chemical and Molecular Simulation Study. J. Am. Chem. Soc. 127, 14250–14256 (2005). 16218619 10.1021/ja0526719

[b3] Thuéry P. & Masci B. Uranyl-Organic Frameworks with 1,2,3,4-Butanetetracarboxylate and 1,2,3,4-Cyclobutanetetracarboxylate Ligands. Cryst. Growth Des. 8, 3430–3436 (2008).

[b4] Xu X.-T. *et al.* UO_2_^2+^**-**amino hybrid materials: structural variation and photocatalysis properties. Cryst. Eng. Comm. 17, 642–652 (2015).

[b5] Jin N., Ibrahim M., Spiro T. G. & Groves J. T. Trans-dioxo Manganese(V) Porphyrins. J. Am. Chem. Soc. 129, 12416–12417 (2007). 17887684 10.1021/ja0761737 PMC2773832

[b6] Spencer L. P., Yang P., Scott B. L., Batista E. R. & Boncella J. M. Uranium(VI) Bis(imido) Chalcogenate Complexes: Synthesis and Density Functional Theory Analysis. Inorg. Chem. 48, 2693–2700 (2009). 19216556 10.1021/ic802212m

[b7] Karmel I. S. R., Fridman N., Tamm M. & Eisen M. S. Mono(imidazolin-2-iminato) Actinide Complexes: Synthesis and Application in the Catalytic Dimerization of Aldehydes, J. Am. Chem. Soc. 136, 17180–17192 (2014). 25393398 10.1021/ja5091436

[b8] Bharara M. S., Strawbridge K., Vilsek J. Z., Bray T. H. & Gorden A. E. V. Novel Dinuclear Uranyl Complexes with Asymmetric Schiff Base Ligands: Synthesis, Structural Characterization, Reactivity, and Extraction Studies. Inorg. Chem. 46, 8309–8315 (2007). 17722915 10.1021/ic7010315

[b9] Yu Z. T., Liao Z. L., Jiang Y. S., Li G. H. & Chen J. S. Water-Insoluble Ag-U-Organic Assemblies with Photocatalytic Activity. Chem. Eur. J. 11, 2642–2650 (2005). 15729678 10.1002/chem.200401189

[b10] Liao Z. L., Li G. D., Bi M. H. & Chen J. S. Preparation, Structures, and Photocatalytic Properties of Three New Uranyl-Organic Assembly Compounds. Inorg. Chim. Acta 47, 4844–4853 (2008). 10.1021/ic800109y 18447330

[b11] Gao X. *et al.* A family of uranyl-aromatic dicarboxylate (pht-, ipa-, tpa-) framework hybrid materials: photoluminescence, surface photovoltage and dye adsorption. Dalton Trans. 44, 11562–11571 (2015). 26038888 10.1039/c5dt01470k

[b12] Corden J. P., Errington W., Moore P. & Wallbridge M. G. H. N,N’-Bis(2-hydroxybenzylidene)-2,2-dimethyl-1,3-propanediamine. Act Cryst. C52, 125–127 (1996).

[b13] Kepert D. L. Aspects of the Stereochemistry of Seven-Coordination. Prog. Inorg. Chem. 25, 41–144 (1979).

[b14] Allen F. H. The Cambridge Structural Database: a quarter of a million crystal structures and rising. Acta Crystallogr. B 58, 380–388 (2002). 10.1107/s0108768102003890 12037359

[b15] Desiraju G. R. & Steiner T. The Weak Hydrogen Bond in Structural Chemistry and Biology, Oxford University Press, Oxford (1999).

[b16] Nour E. M., Taha A. A. & Alnaimi I. S. Infrared and Raman studies of [UO_2_ (salen)(L)](L=H_2_O and CH_3_OH). Inorg. Chim. Acta 141, 139–144 (1988).

[b17] Kolawole G. A. & Patel S. K. The Stereochemistry of Oxovanadium(iv) Complexes derived from Salicylaldehyde and Polymethylenediamines. J. Chem. Soc., Dalton Trans. 1241–1245 (1981).

[b18] Franczyk T. S., Czerwinski K. R. & Raymond K. N. Stereognostic coordination chemistry. 1. The design and synthesis of chelators for the uranyl ion. J. Am. Chem. Soc. 114, 8138–8146 (1992).

[b19] Bharara M. S., Heflin K., Tonks S., Strawbridge K. L. & Gorden A. E. V. Hydroxy- and alkoxy-bridged dinuclear uranyl–Schiff base complexes: hydrolysis, transamination and extraction studies. Dalton Trans. 10, 2966–2973 (2008). 18493632 10.1039/b800469b

[b20] Ulusoy M., Birel O., Sahin O., Buyukgungor O. & Cetinkya B. Structural, spectral, electrochemical and catalytic reactivity studies of a series of N_2_O_2_ chelated palladium(II) complexes. Polyhedron 38, 141–148 (2012).

[b21] Hardwick H. C. *et al.* Structural, spectroscopic and redox properties of uranyl complexes with a maleonitrile containing ligand. Dalton Trans. 40, 5939–5952 (2011). 21526261 10.1039/c0dt01580f

[b22] Vlaisavljevich B., Diaconescu P. L., Lukens W. L., Gagliardi L. Jr. & Cummins C. C. Investigations of the Electronic Structure of Arene-Bridged Diuranium Complexes. Organometallics 32, 1341–1352 (2013).

[b23] Azam M. *et al.* Novel uranyl(VI) complexes incorporating propylene-bridged salen-type N_2_O_2_-ligands: a structural and computational approach. Dalton Trans. 44, 568–577 (2015). 25380389 10.1039/c4dt02112f

[b24] Tenderholt A. “QMForge: A Program to Analyze Quantum Chemistry Calculations”, Version 2.3.2, Stanford University, Stanford, CA, 2007, http://qmforge.sourceforge.net.

[b25] Velde G. T. *et al.* Chemistry with ADF. J. Comput. Chem. 22, 931–967 (2001).

[b26] Zhai X.-S., Zheng Y.-Q., Lin J.-L. & Xu W. Four new dinuclear uranyl complexes based on *p*- and *m*-toluic acid: Syntheses, structures, luminescent and photocatalytic properties. Inorg. Chim. Acta 423, 1–10 (2014).

[b27] Yu Z. T., Liao Z. L., Jiang Y. S., Li G. H. & Chen J. S. Water-Insoluble Ag–U–Organic Assemblies with Photocatalytic Activity. Chem. Eur. J. 11, 2642–2650 (2005). 15729678 10.1002/chem.200401189

[b28] Volkovich V. A., Griffiths T. R., Fray D. J. & Thied R. C. The electronic spectra of alkali metal uranates and band assignments: an analysis of their diffuse reflectance spectra. Phys. Chem. Chem. Phys. 3, 5182–5191 (2001).

[b29] Guan Q. L., Bai F. Y., Xing Y. H., Lie J. & Zhang H. Z. Unexpected cis-dioxido uranyl carboxylate compound: Synthesis, characterization and photocatalytic activity of uranyl-succinate complexe. Inorg. Chem. Commun. 95, 36–40 (2015).

[b30] Li Y., Su J., Mitchell E., Zhang G.-Q. & Li J. Photocatalysis with visible-light-active uranyl complexes. Sci. China Chem. 56, 1671–1681 (2013).

[b31] Yu Z.-T., Liao Z.-L., Jiang Y.-S., Li G.-H. & Chen J.-S. Water-Insoluble Ag–U–Organic Assemblies with Photocatalytic Activity. Chem. Eur. J. 11, 2642–2650 (2005). 15729678 10.1002/chem.200401189

[b32] Horikoshi S., Saitou A., Hidaka H. & Serpone N. Environmental Remediation by an Integrated Microwave/UV Illumination Method. V. Thermal and Nonthermal Effects of Microwave Radiation on the Photocatalyst and on the Photodegradation of Rhodamine-B under UV/Vis Radiation. Environ. Sci. Technol. 37, 5813–5822 (2003). 14717200 10.1021/es030326i

[b33] Wang C.-W., Li J.-R., Lv X.-L., Zhnag Y.-Q. & Guo G. Photocatalytic organic pollutants degradation in metal–organic frameworks. Energy Environ. Sc. 7, 2831–2867 (2014).

[b34] Brachmann A., Geipel G., Bernhard G. & Ntische H. Study of uranyl(VI) malonate complexation by time resolved laser-induced fluorescence spectroscopy (TRLFS).Radiochim. Acta 90, 147–153 (2002).

[b35] Lauren A. B. & Christopher L. C. Crystal Engineering with the Uranyl Cation II. Mixed Aliphatic Carboxylate/Aromatic Pyridyl Coordination Polymers: Synthesis, Crystal Structures, and Sensitized Luminescence. Cryst. Growth Des. 6, 2248–2259 (2006).

[b36] Frisch M. J. *et al.* Gaussian 09, revision B.01; Gaussian, Inc.: Wallingford, CT, (2009).

[b37] Becke A. D. Density‐functional thermochemistry. III. The role of exact exchange. J. Chem. Phys. 98, 5648–5652 (1993).

[b38] Lee C. T., Yang W. T. & Parr R. G. Development of the Colle-Salvetti correlation-energy formula into a functional of the electron density. Phys. Rev. B. 37, 785–789 (1988). 10.1103/physrevb.37.785 9944570

[b39] Stephens P. J., Devlin F. J., Chabalowski C. F. & Frisch M. J. Ab Initio Calculation of Vibrational Absorption and Circular Dichroism Spectra Using Density Functional Force Fields. J. Phys. Chem. 98, 11623–11627 (1994).

[b40] Küchle W., Dolg M., Stoll H. & Preuss H. Molecular Physics: An International Journal at the Interface between Chemistry and Physics. Mol. Phys. 74, 1245–1263 (1991).

[b41] Küchle W., Dolg M., Stoll H. & Preuss H. Energy‐adjusted pseudopotentials for the actinides. Parameter sets and test calculations for thorium and thorium monoxide. J. Chem. Phys. 100, 7535–7542 (1994).

[b42] Stratmann R. E., Scuseria G. E. & Frisch M. J. An efficient implementation of time-dependent density-functional theory for the calculation of excitation energies of large molecules. J. Chem. Phys., 109, 8218–8224 (1998).

[b43] Glendening E., Reed A., Carpenter J. & Weinhold F. University of Wisconsin: Madison (1998).

[b44] Reed A. E., Curtiss L. A. & Weinhold F. Intermolecular interactions from a natural bond orbital, donor-acceptor viewpoint. Chem. Rev. 88, 899–926 (1988).

[b45] Perdew J. P. Density-functional approximation for the correlation energy of the inhomogeneous electron gas. Phys. Rev. B 33, 8822–8824 (1986). 10.1103/physrevb.33.8822 9938299

[b46] Velde G. T. *et al.* Chemistry with ADF. J. Comput. Chem. 22, 931–967 (2001).

[b47] X-RED. Version 1.18. STOE & Cie GmbH, Darmstadt, Germany (1999).

[b48] Sheldrick G. M. A short history of *SHELX*. Acta Crystallogr. A64, 112–122 (2008). 10.1107/S0108767307043930 18156677

