# Supplementary material for: Structural elucidation and physicochemical properties of mononuclear Uranyl(VI) complexes incorporating dianionic units
Source: Sci Rep. 2016 Sep 6;6:32898. doi: 10.1038/srep32898 (PMC5011772; doi:10.1038/srep32898)

Structural elucidation and physicochemical properties of mononuclear Uranyl(VI) complexes incorporating dianionic units

Mohammad Azam[1*], Gunasekaran Velmurugan[2], Saikh Mohammad Wabaidur[1], Agata Trzesowska-Kruszynska[3], Rafal Kruszynski[3], Saud I. Al-Resayes[1], Zeid A. Al-Othman[1], Ponnambalam Venuvanalingam[2]

[1]*Department of Chemistry, College of Science, King Saud University, P. O. Box 2455, Riyadh 11451, KSA*
[2]*School of Chemistry, Bharathidasan University, Tiruchirappalli – 620024, Tamil Nadu, INDIA*
[3]*Institute of General and Ecological Chemistry, Lodz University of Technology, Zeromskiego 116, 90-924, Lodz, Poland*

[*]For Correspondence: Email: azam_res@yahoo.com

Supplementary Files

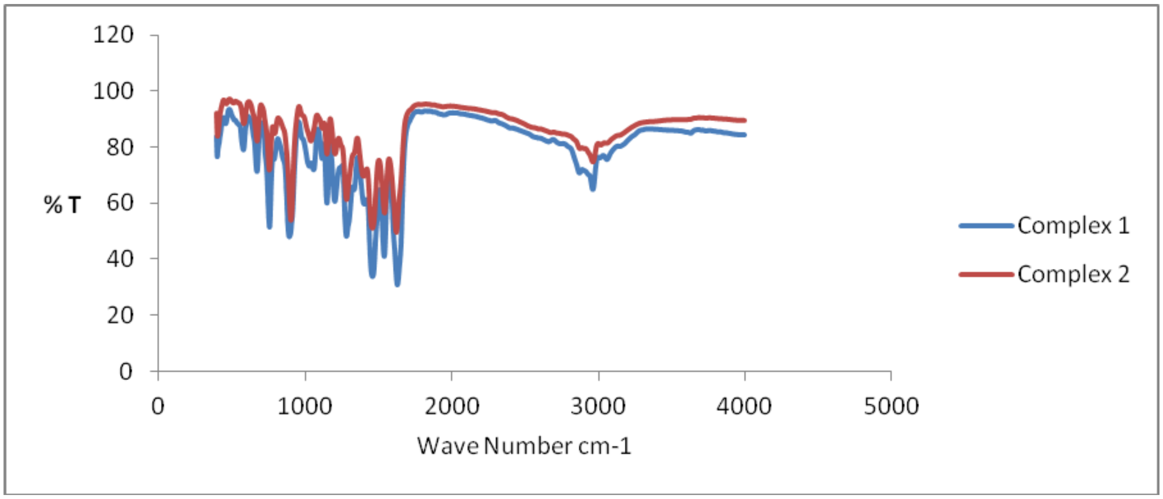

Fig S1: FTIR spectra of uranyl complexes

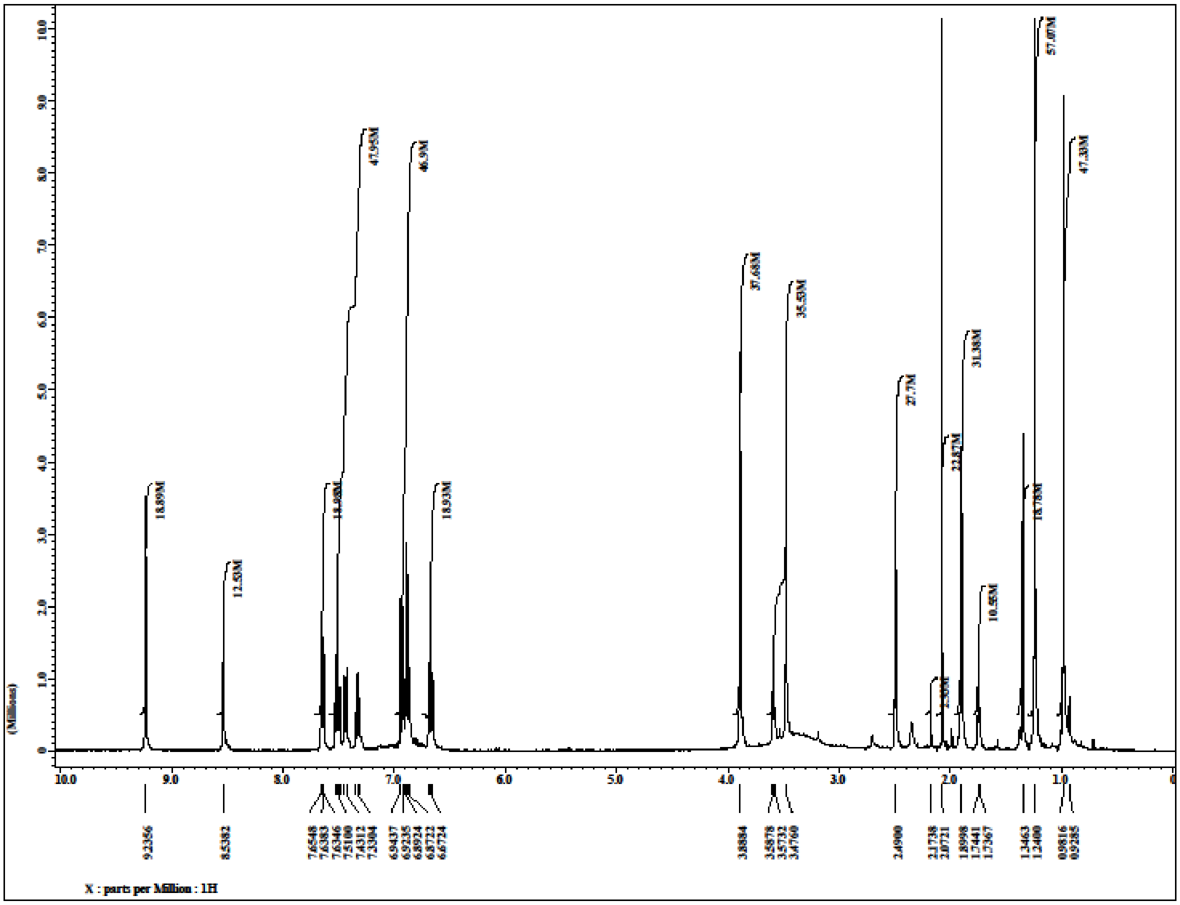

Fig S2: ^{1}H NMR spectrum of complex 1 at r.t. in d_6-DMSO

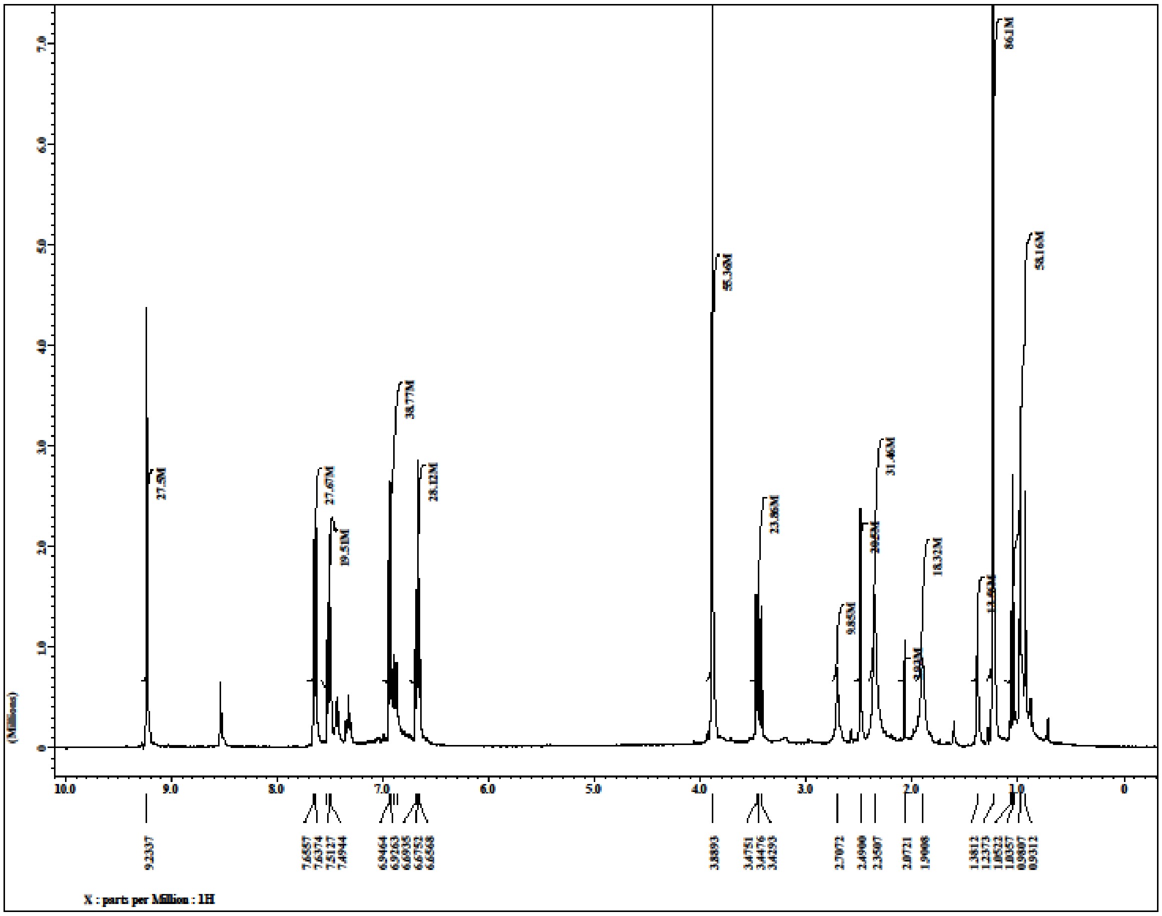

Fig S3: ^{1}H NMR spectrum of Complex 2 at r.t. in d_6-DMSO

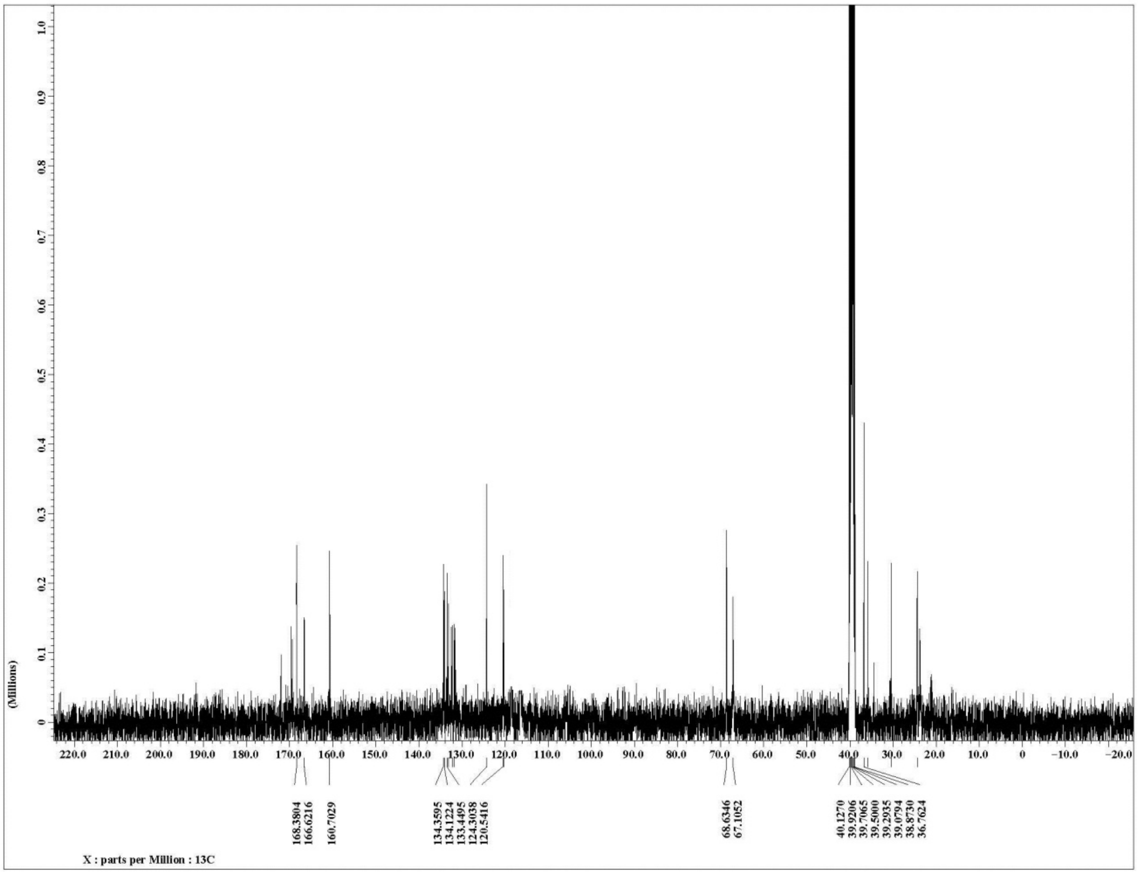

Fig S4: ^{13}C NMR spectrum of Complex 1 at r.t. in d_6-DMSO

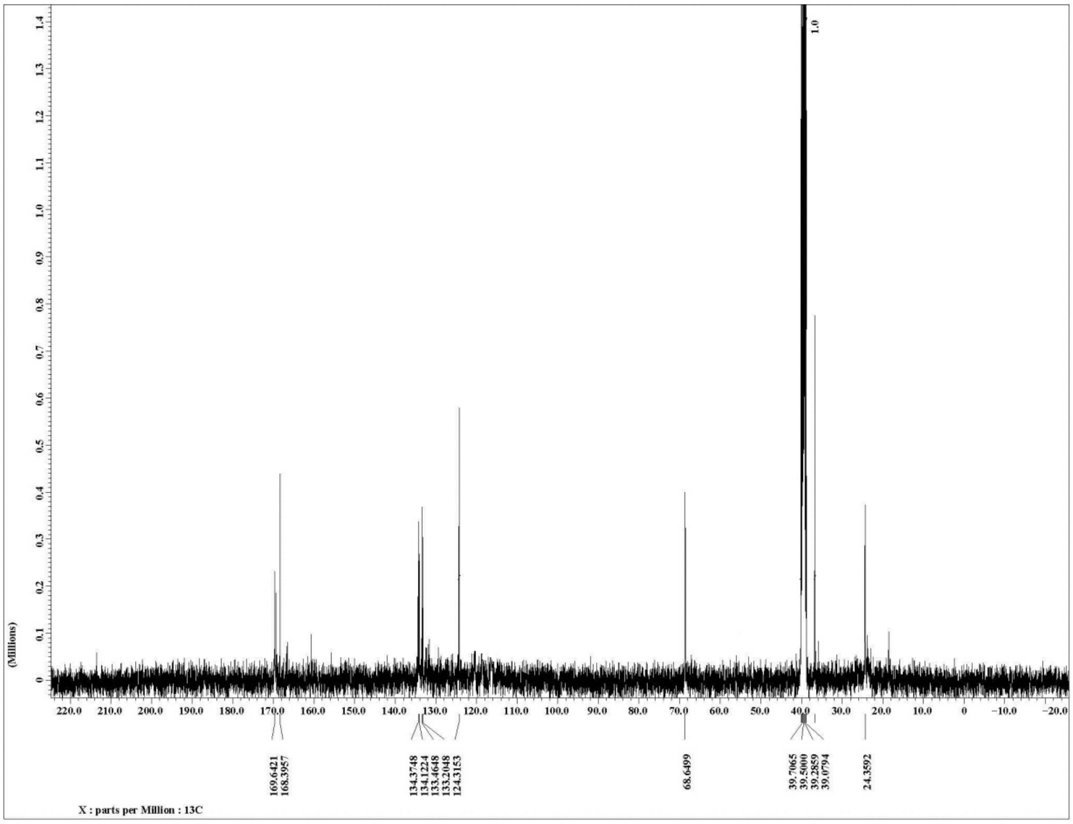

Fig S5: ^{13}C NMR complex 2 at r.t. in d_6-DMSO

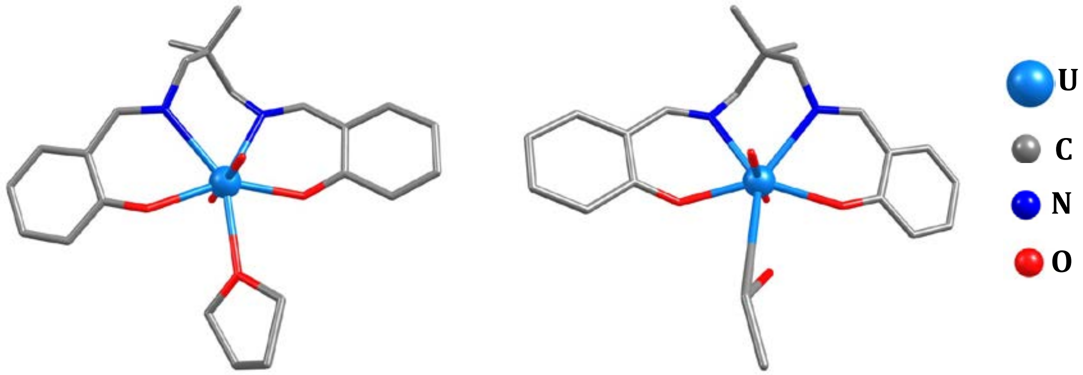

Complex1 **Complex 2**

Fig. S6. Optimized structures of complex [UO_2(L)THF] (1) and [UO_2(L)C_2H_5OH] (2).

Complex1

Complex 2

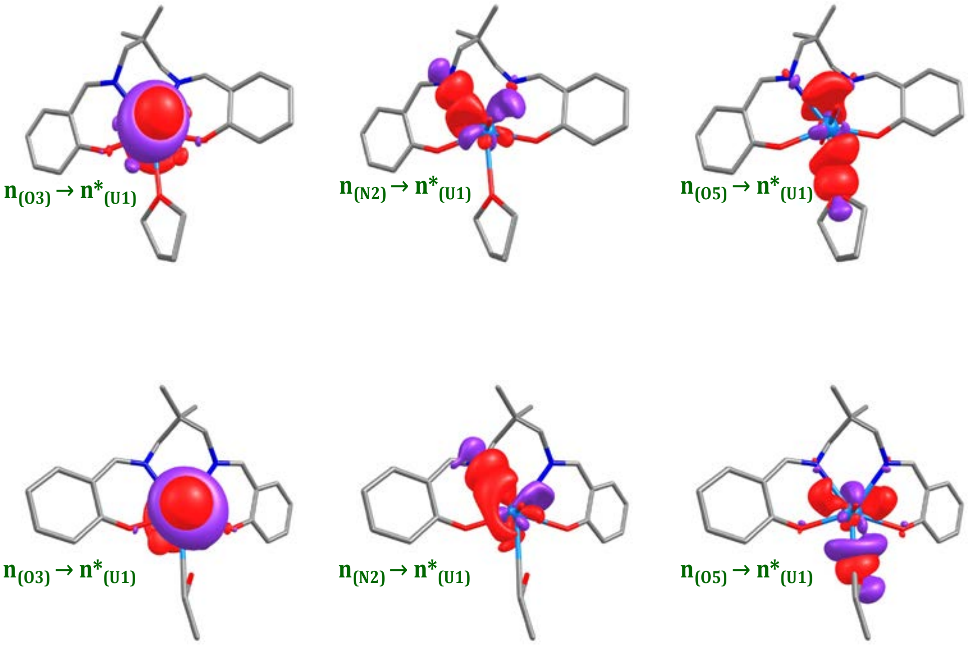

Fig. S7. Ground state stabilizing interactions of complex [UO_2(L)THF] (1) and [UO_2(L)C_2H_5OH] (2).

Table S1. Selected structural parameters for complex 1 and 2 (Distances are given in Å, Angles in ˚)

**Complex 1**			**Complex 2**		
**Bond Parameters**	**Exp**	**Cal**	**Bond Parameters**	**Exp**	**Cal**
U1-O1	2.247	2.269	U1-O1	2.305	2.315
U1-O2	2.247	2.269	U1-O2	2.230	2.224
U1-O3	1.793	1.786	U1-O3	1.782	1.786
U1-O4	1.793	1.786	U1-O4	1.782	1.782
U1-O5	2.484	2.544	U1-O5	2.447	2.580
U1-N1	2.587	2.629	U1-N1	2.565	2.594
U1-N2	2.587	2.629	U1-N2	2.573	2.598
O2-C15	1.311	1.307	O2-C15	1.324	1.310
O1-C1	1.311	1.307	O1-C1	1.330	1.312
C14-C15	1.419	1.430	C14-C15	1.406	1.430
C1-C2	1.419	1.430	C1-C2	1.422	1.431
C14-C13	1.450	1.446	C14-C13	1.460	1.447
C2-C7	1.450	1.446	C2-C7	1.456	1.447
N2-C13	1.283	1.292	N2-C13	1.284	1.293
N1-C7	1.283	1.292	N1-C7	1.283	1.292
N2-C12	1.460	1.463	N2-C12	1.468	1.464
N1-C8	1.460	1.463	N1-C8	1.471	1.465
O2-U1-O5	74.62	75.19	O2-U1-O5	75.36	81.51
O1-U1-O5	74.62	75.19	O1-U1-O5	76.46	66.60
O4-U1-O5	91.50	91.07	O4-U1-O5	88.13	95.96
O3-U1-O5	91.50	91.06	O3-U1-O5	93.70	87.45
O4-U1-O3	177.00	177.87	O4-U1-O3	177.05	176.37
U1-O5-C20	125.12	124.56	U1-O5-C20	125.09	122.68
U1-N1-C7	126.88	127.58	U1-N1-C7	125.99	127.30
U1-N2-C13	126.88	127.58	U1-N2-C13	125.89	126.85

Table S2: Molecular Orbital Composition (%) for Complexes 1 and 2

	Complex 1 $[UO_2(L)THF]$				Complex 2 $[UO_2(L)C_2H_5OH]$				Complex 3 [ref. 23] $[UO_2(L)CH_3OH]$			
	U	2 ("O")	THF	L	U	2 ("O")	EtOH	L	U	2 ("O")	CH_3OH	L
HOMO-3	0.49	0.07	0.25	99.19	0.53	0.12	0.37	98.98	0.55	0.20	0.42	98.83
HOMO-2	0.29	0.55	0.04	99.10	0.31	0.53	0.03	99.14	0.34	0.45	0.07	99.14
HOMO-1	3.68	3.54	0.11	92.67	3.98	2.86	0.07	93.09	3.67	2.93	0.08	93.32
HOMO	5.67	1.68	0.16	92.49	5.24	1.85	0.08	92.84	5.21	1.72	0.05	93.02
LUMO	82.79	0.07	0.30	16.84	81.88	0.12	0.20	17.81	81.67	0.12	0.17	18.04
LUMO+1	87.87	0.16	0.77	11.21	90.38	0.16	0.65	8.81	90.17	0.16	0.62	9.05
LUMO+2	90.27	0.09	1.39	8.25	89.23	0.15	1.39	9.24	89.54	0.12	1.43	8.92
LUMO+3	82.66	0.23	1.12	15.99	84.00	0.14	1.13	14.72	83.69	0.16	1.07	15.08

Table S3. Energy Decomposition ($kJ\ mol^{-1}$) for Complexes 1 and 2

	Complex 1	Complex 2
Pauli repulsion	168.57	592.94
Electrostatic interaction	-145.53	-396.65
Total steric interaction	23.04	196.28
Orbital interactions	-76.30	-206.53
Total bonding energy	-73.71	-28.91

checkCIF/PLATON report

Structure factors have been supplied for datablock(s) maz_ns09

THIS REPORT IS FOR GUIDANCE ONLY. IF USED AS PART OF A REVIEW PROCEDURE FOR PUBLICATION, IT SHOULD NOT REPLACE THE EXPERTISE OF AN EXPERIENCED CRYSTALLOGRAPHIC REFEREE.

No syntax errors found.   CIF dictionary   Interpreting this report

Datablock: maz_ns09

```
Bond precision:  C-C = 0.0078 A          Wavelength=1.54178

Cell:           a=17.2330(15)    b=13.5930(11)       c=11.8320(9)
                alpha=90         beta=125.429(12)    gamma=90
Temperature:    100 K

                Calculated                 Reported
Volume          2258.4(5)                  2258.4(5)
Space group     C 2/c                      C 2/c
Hall group      -C 2yc                     -C 2yc
Moiety formula  C23 H28 N2 O5 U            C23 H28 N2 O5 U
Sum formula     C23 H28 N2 O5 U            C23 H28 N2 O5 U
Mr              650.50                     650.50
Dx,g cm-3       1.913                      1.913
Z               4                          4
Mu (mm-1)       20.537                     20.537
F000            1248.0                     1248.0
F000'           1234.42
h,k,lmax        21,16,14                   21,16,14
Nref            2246                       2242
Tmin,Tmax       0.236,0.171                0.185,0.221
Tmin'           0.105

Correction method= # Reported T Limits: Tmin=0.185 Tmax=0.221
AbsCorr = NUMERICAL

Data completeness= 0.998          Theta(max)= 72.370

R(reflections)= 0.0342( 2242)     wR2(reflections)= 0.0772( 2242)

S = 1.093                  Npar= 144
```

The following ALERTS were generated. Each ALERT has the format
test-name_ALERT_alert-type_alert-level.
Click on the hyperlinks for more details of the test.

Alert level C

```
PLAT220_ALERT_2_C Non-Solvent Resd 1   C   Ueq(max)/Ueq(min) Range        3.6 Ratio
```

Alert level G

```
PLAT128_ALERT_4_G Alternate Setting for Input Space Group     C2/c       I2/a Note
PLAT180_ALERT_4_G Check Cell Rounding: # of Values Ending with 0 =          3 Note
```

```
   0 ALERT level A = Most likely a serious problem - resolve or explain
   0 ALERT level B = A potentially serious problem, consider carefully
   1 ALERT level C = Check. Ensure it is not caused by an omission or oversight
   2 ALERT level G = General information/check it is not something unexpected

   0 ALERT type 1 CIF construction/syntax error, inconsistent or missing data
   1 ALERT type 2 Indicator that the structure model may be wrong or deficient
   0 ALERT type 3 Indicator that the structure quality may be low
   2 ALERT type 4 Improvement, methodology, query or suggestion
   0 ALERT type 5 Informative message, check
```

It is advisable to attempt to resolve as many as possible of the alerts in all categories. Often the minor alerts point to easily fixed oversights, errors and omissions in your CIF or refinement strategy, so attention to these fine details can be worthwhile. In order to resolve some of the more serious problems it may be necessary to carry out additional measurements or structure refinements. However, the purpose of your study may justify the reported deviations and the more serious of these should normally be commented upon in the discussion or experimental section of a paper or in the "special_details" fields of the CIF. checkCIF was carefully designed to identify outliers and unusual parameters, but every test has its limitations and alerts that are not important in a particular case may appear. Conversely, the absence of alerts does not guarantee there are no aspects of the results needing attention. It is up to the individual to critically assess their own results and, if necessary, seek expert advice.

Publication of your CIF in IUCr journals

A basic structural check has been run on your CIF. These basic checks will be run on all CIFs submitted for publication in IUCr journals (*Acta Crystallographica*, *Journal of Applied Crystallography*, *Journal of Synchrotron Radiation*); however, if you intend to submit to *Acta Crystallographica Section C* or *E* or *IUCrData*, you should make sure that full publication checks are run on the final version of your CIF prior to submission.

Publication of your CIF in other journals

Please refer to the *Notes for Authors* of the relevant journal for any special instructions relating to CIF submission.

PLATON version of 06/05/2016; check.def file version of 05/05/2016

Datablock maz_ns09 - ellipsoid plot

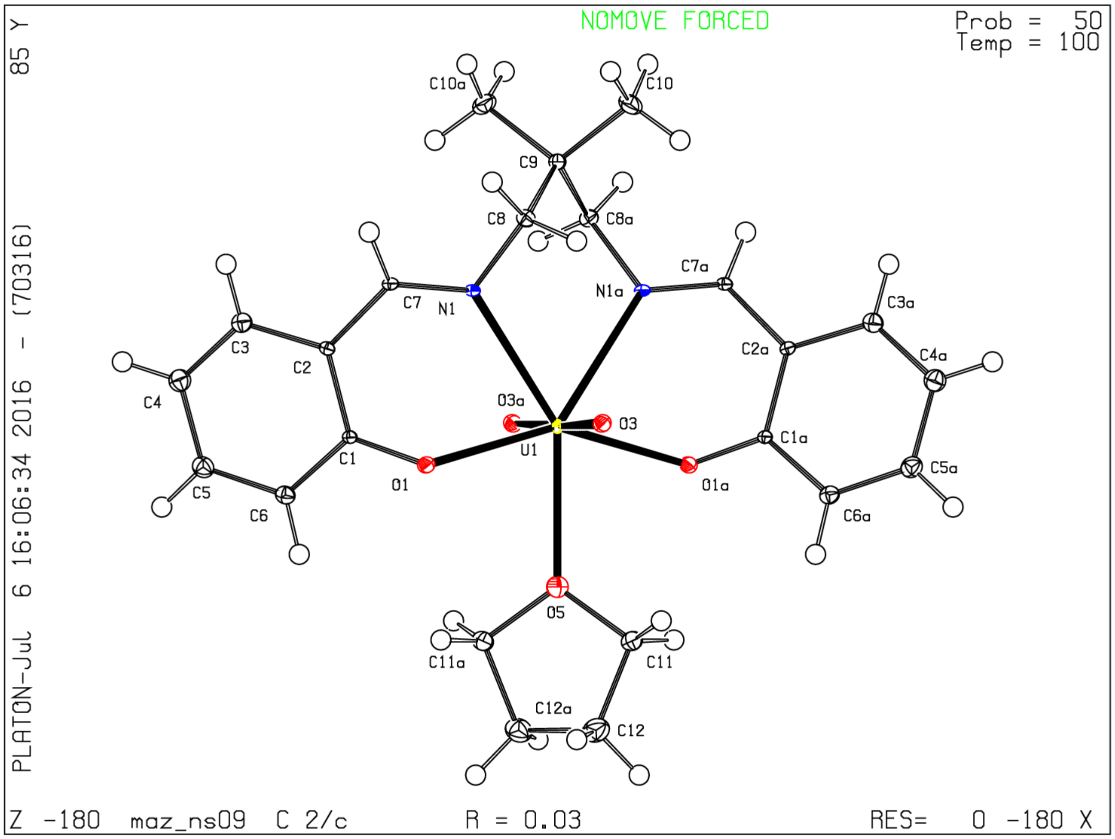

checkCIF/PLATON report

Structure factors have been supplied for datablock(s) maz_ns11

THIS REPORT IS FOR GUIDANCE ONLY. IF USED AS PART OF A REVIEW PROCEDURE FOR PUBLICATION, IT SHOULD NOT REPLACE THE EXPERTISE OF AN EXPERIENCED CRYSTALLOGRAPHIC REFEREE.

No syntax errors found. CIF dictionary Interpreting this report

Datablock: maz_ns11

```
Bond precision:  C-C = 0.0075 A          Wavelength=1.54178

Cell:           a=11.4887(14)    b=12.522(2)         c=15.1669(17)
                alpha=90         beta=92.092(10)     gamma=90
Temperature:    100 K

                Calculated                 Reported
Volume          2180.5(5)                  2180.5(5)
Space group     P 21/n                     P 21/n
Hall group      -P 2yn                     -P 2yn
Moiety formula  C21 H26 N2 O5 U            C21 H26 N2 O5 U
Sum formula     C21 H26 N2 O5 U            C21 H26 N2 O5 U
Mr              624.47                     624.47
Dx,g cm-3       1.902                      1.902
Z               4                          4
Mu (mm-1)       21.238                     21.238
F000            1192.0                     1192.0
F000'           1178.34
h,k,lmax        14,15,18                   14,15,18
Nref            4323                       4309
Tmin,Tmax       0.217,0.154                0.161,0.203
Tmin'           0.097

Correction method= # Reported T Limits: Tmin=0.161 Tmax=0.203
AbsCorr = NUMERICAL

Data completeness= 0.997          Theta(max)= 72.470

R(reflections)= 0.0328( 4274)     wR2(reflections)= 0.0873( 4309)

S = 1.089                 Npar= 265
```

The following ALERTS were generated. Each ALERT has the format
test-name_ALERT_alert-type_alert-level.
Click on the hyperlinks for more details of the test.

Alert level B

PLAT220_ALERT_2_B Non-Solvent Resd 1 C Ueq(max)/Ueq(min) Range 6.1 Ratio

Author Response: The compound 2 (maz_ns11) contains ethanol molecule directly coordinating to the central uranium ion. Around this ligand is small empty void, what allows both, larger thermal motion of the ethanol molecule carbon atoms, and slightly different location of the molecules in the subsequent unit cells. This leads to enlargement of displacement ellipsoids of the ethanol molecule atoms, in comparison to the uranium ion and the imine ligand. This effect is not observed in the compound 1 (maz_ns09) as the tetrahydrofuran molecule is more bulky than the ethanol molecule.

PLAT230_ALERT_2_B Hirshfeld Test Diff for O5 -- C20 .. 11.0 s.u.

Author Response: The compound 2 (maz_ns11) contains ethanol molecule directly coordinating to the central uranium ion. Around this ligand is small empty void, what allows both, larger thermal motion of the ethanol molecule carbon atoms, and slightly different location of the molecules in the subsequent unit cells. This leads to enlargement of displacement ellipsoids of the ethanol molecule carbon atoms, in comparison to its oxygen atom. The bonding of the oxygen atom to the heavy uranium ion (via coordination bond) and to relatively light (in comparison to uranium) carbon atom (via covalent bond) leads to different directions of displacement of the carbon and oxygen atoms.

Alert level C

PLAT222_ALERT_3_C Non-Solvent Resd 1 H Uiso(max)/Uiso(min) Range 5.1 Ratio

---

Alert level G

PLAT007_ALERT_5_G Number of Unrefined Donor-H Atoms .... 1 Report

---

0 **ALERT level A** = Most likely a serious problem - resolve or explain
2 **ALERT level B** = A potentially serious problem, consider carefully
1 **ALERT level C** = Check. Ensure it is not caused by an omission or oversight
1 **ALERT level G** = General information/check it is not something unexpected

0 ALERT type 1 CIF construction/syntax error, inconsistent or missing data
2 ALERT type 2 Indicator that the structure model may be wrong or deficient
1 ALERT type 3 Indicator that the structure quality may be low
0 ALERT type 4 Improvement, methodology, query or suggestion
1 ALERT type 5 Informative message, check

It is advisable to attempt to resolve as many as possible of the alerts in all categories. Often the minor alerts point to easily fixed oversights, errors and omissions in your CIF or refinement strategy, so attention to these fine details can be worthwhile. In order to resolve some of the more serious problems it may be necessary to carry out additional measurements or structure refinements. However, the purpose of your study may justify the reported deviations and the more serious of these should normally be commented upon in the discussion or experimental section of a paper or in the "special_details" fields of the CIF. checkCIF was carefully designed to identify outliers and unusual parameters, but every test has its limitations and alerts that are not important in a particular case may appear. Conversely, the absence of alerts does not guarantee there are no aspects of the results needing attention. It is up to the individual to critically assess their own results and, if necessary, seek expert advice.

Publication of your CIF in IUCr journals

A basic structural check has been run on your CIF. These basic checks will be run on all CIFs submitted for publication in IUCr journals (*Acta Crystallographica*, *Journal of Applied Crystallography*, *Journal of Synchrotron Radiation*); however, if you intend to submit to *Acta Crystallographica Section C* or *E* or *IUCrData*, you should make sure that full publication checks are run on the final version of your CIF prior to submission.

Publication of your CIF in other journals

Please refer to the *Notes for Authors* of the relevant journal for any special instructions relating to CIF submission.

PLATON version of 06/05/2016; check.def file version of 05/05/2016

Datablock maz_ns11 - ellipsoid plot

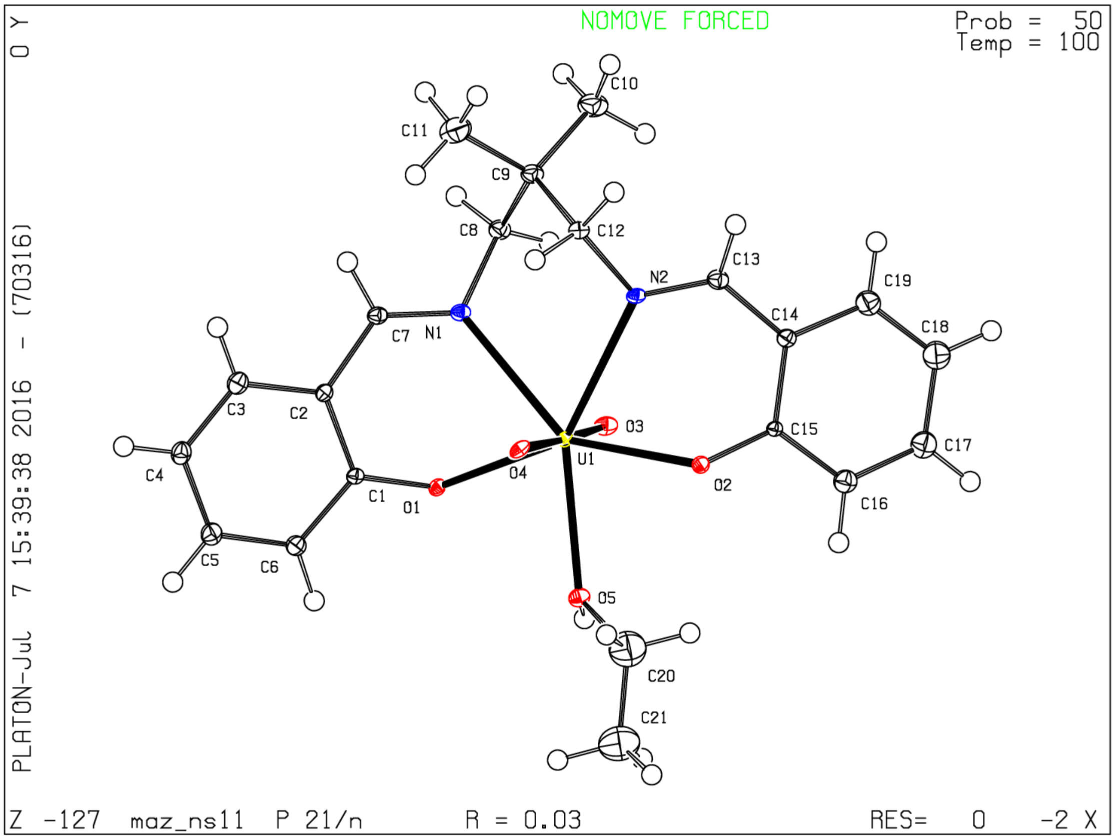

Supplement: Supplementary Information [file srep32898-s1.pdf]
